# Growth Hormone and IGF1 Actions in Kidney Development and Function

**DOI:** 10.3390/cells10123371

**Published:** 2021-11-30

**Authors:** Evgenia Gurevich, Yael Segev, Daniel Landau

**Affiliations:** 1 Department of Nephrology, Schneider Children’s Medical Center of Israel, 14 Kaplan Street, Petach Tikva 4920235, Israel; gurevichjeny@gmail.com; 2 Shraga Segal Department of Microbiology and Immunology, Ben Gurion University, Beer Sheva 8410501, Israel; yaelse@bgu.ac.il; 3 Sackler School of Medicine, Tel Aviv University, P.O. Box 39040, Tel Aviv 6997801, Israel

**Keywords:** growth hormone, insulin-like growth factor 1, growth hormone receptor, receptor signaling, diabetic nephropathy, chronic kidney disease, kidney hypertrophy

## Abstract

Growth hormone (GH) exerts multiple effects on different organs including the kidneys, either directly or via its main mediator, insulin-like-growth factor-1 (IGF-1). The GH/IGF1 system plays a key role in normal kidney development, glomerular hemodynamic regulation, as well as tubular water, sodium, phosphate, and calcium handling. Transgenic animal models demonstrated that GH excess (and not IGF1) may lead to hyperfiltration, albuminuria, and glomerulosclerosis. GH and IGF-1 play a significant role in the early development of diabetic nephropathy, as well as in compensatory kidney hypertrophy after unilateral nephrectomy. Chronic kidney disease (CKD) and its complications in children are associated with alterations in the GH/IGF1 axis, including growth retardation, related to a GH-resistant state, attributed to impaired kidney postreceptor GH-signaling and chronic inflammation. This may explain the safety of prolonged rhGH-treatment of short stature in CKD.

## 1. Introduction

Most animals must undergo a transition from maternal environment to independent life through processes of growth and maturation. Important hormonal regulators of childhood growth are growth hormone (GH), insulin-like growth factor 1 (IGF1), sex steroids, and thyroid hormone. GH and IGF1 are part of an axis, which is essential for bone and organs growth. The kidneys express both GH as well as IGF1 receptors, and are one of the key target organs for these hormones’ actions. This review concentrated on the roles of these hormones in physiological and pathological kidney conditions.

## 2. Normal GH-IGF1 Axis and Physiology

GH is produced by somatotroph cells of the anterior pituitary and secreted in a pulsatory way under the positive control of hypothalamic GH-releasing hormone (GHRH) and the negative control of somatostatin [1]. The response to GHRH is mediated via GH-releasing hormone receptor (GHRHR), a G protein–coupled receptor (GPCR) expressed specifically in somatotrophs [2]. Other factors such as insulin-like growth factor (IGF1), neuropeptide Y, and hyperglycemia inhibit GH secretion, and hypoglycemia, thyroxine, ghrelin, klotho, and glucocorticoids stimulate GH secretion [3].

GH acts by binding to GH receptor (GHR) to stimulate, among other genes, the synthesis of insulin-growth factor-1 (IGF1). The bioavailability of GH is regulated by GH-binding protein (GHBP), which is the extracellular part of GHR. Intracellular signal transduction after GH binding to its receptor requires the activation of Janus-associated kinase 2 (JAK2) [4], which stimulates phosphorylation of signal transducer and activator of transcription (STAT) proteins MAPK and PI3K. STAT proteins migrate to the nucleus, activating, among others, gene transcription of IGF1, the main mediator of GH action. In addition, suppressors of cytokine signaling (SOCS) are activated, which dephosphorylate STAT, leading to a negative feedback action on GH [5]. Circulating IGF1 suppresses pituitary GH secretion in a negative feedback loop. IGF1 is synthesized mostly in the liver, but also in peripheral tissues under GH regulation, although nutrition, insulin, thyroid, and sex hormones also affect its expression [6]. The effects of IGF1 are mediated by the type 1 IGF receptor (IGF1R) in a signaling pathway similar to insulin/insulin receptor (IR). IGF1R and IR share amino acid identity, and can be activated both by insulin, IGF1, and IGF2. [7]. IGF1R is a membrane-bound tyrosine kinase heterotetramer, and its activation leads to autophosphorylation of tyrosine residues, leading to signal transduction [8]. The bioactivity of circulating IGF1 is modulated by IGF-binding proteins (IGFBPs 1-6), which facilitate its stability in serum and extracellular matrices. Most IGFs in serum are bound to IGFBP and the acid-labile subunit (ALS), a protein that stabilizes IGF [9,10], and this complex serves as reservoir of IGFs, keeping serum concentration of free IGFs constant. Plasma concentration of IGFBP3 and ALS are also increased by GH, similar to IGF1.

## 3. GH-IGF1: Axis or Independent Functions?

Whereas GH is only synthesized in pituitary, GHR and IGF1 are expressed in many tissues including the kidneys. Originally GH action was thought to be mediated only through IGF1, called somatomedin, without any direct effects (“somatomedin theory”) [11]. Later, “dual effector hypothesis” suggested that GH also acts directly to promote cell differentiation, independent of IGF1 [12,13,14]. Concentrating on kidneys as one of the target organs for both GH and IGF-1, GH treatment increased kidney IGF1 mRNA levels in hypophysectomized rats, confirming local renal IGF1 production [15]. IGF1 levels are higher in renal venous blood than in renal arterial blood, suggesting significant renal IGF1 biosynthesis [16]. Evidence for a direct IGF1 action in the kidney also comes from studies showing that prolonged treatment with recombinant human (rh) IGF1 increased kidney size in hypophysectomized rats [17] and enhanced the glomerular filtration rate (GFR) in healthy men [18].

## 4. Observations from Knockout and Transgenic Animals

Animal models of gene inactivation, as well as pathophysiological models, provide important data on mechanisms and role of GH/IGF1 in renal organogenesis. Evidence of both dependent and independent functions of GH and IGF1 on the kidney come from genetically engineered animal models (see Table 1 and Table 2). Examples of the knockout models, where mutations were introduced in every step of the axis (GHRH → GH → GHR/GHBP → JAK2 → STAT5 → IGF1 → IGF1R), followed by the transgenic models, overexpressing genes along this axis, are discussed here.

Biallelic mutation in *GHRH* causes isolated growth hormone deficiency due to impaired GH secretion in anterior hypophysis [2].

GH knockout mice (GH −/−), which show no circulating GH, also show disproportionally reduced kidney weight compared with wild-type mice, even after correction for reduced body weight [19].

GHR/GHPB knockout mice lack functional GH receptors and exhibit GH resistance manifested by decrease in circulating IGF1 levels and growth retardation, starting later after birth. These mice also have disproportionally small kidneys [20].

Germline deletion of Jak2 (downstream of GHR, but also of other hormones and cytokines) in mice resulted in embryonic lethality due to a lack of hematopoiesis [21]. Homozygous mutation in the gene for STAT5 resulted in IGF-1 deficiency and growth hormone insensitivity, indicating impaired postreceptor signaling for GH. It leads to abnormal postnatal growth, facial dysmorphism, and markedly reduced serum concentrations of IGF-1, IGFBP-3, and acid-labile subunit, and immunodeficiency [22]. The latter seems to be due to the importance of both JAK2 and STAT5 not just in mediating GH signals, but also other cytokines involved in immune as well as hematopoetic regulation, such as the erythropoietin receptor [23]. STAT5 knockout mice died perinatally, and 1–2% of survivors were dwarf, with anemia and immunodeficiency [24].

IGF1 knockout mice have severe growth retardation, deficiencies in bone and muscle development, infertility, and lethal respiratory failure due to lung hypoplasia, highlighting the importance of GH/IGF1 axis in different tissues development. Their kidneys are proportionally small with decreased glomerular size and nephron number [25,26].

Prenatal IGF1R knockout embryos exhibit growth retardation and generalized developmental abnormalities, comprising hypoplasia, altered central nervous systems, abnormal skin formation, delayed bone development, reduced pancreatic beta-cells, failure of testicular determination, lung immaturity, and cochlear defects [27]. As IGF1R is closely related to the IR, partly sharing amino acid identity, increased IGF2–mediated IR signaling can rescue mouse embryonic development to prevent dwarfism in IGF1R knockout mice [28].

Mice with homozygous null mutations in *Igf1r* had normal embryonic development but had low weight and died soon after birth, whereas heterozygous mice had normal growth up to the weaning period, followed by a significant reduction in weight gain and development of insulin resistance [29]. Therefore, the phenotype of the knockout animal is more severe as the location of the affected gene is more distal along the signaling pathway, as described for example for other pathways where the kidneys are a target organ [30].

Excessive GH levels are associated with renal hypertrophy in humans and rodents [31]. Transgenic mice overexpressing the GH gene exhibit excessive GH and IGF1 concentrations, resulting in a giant phenotype and organomegaly, including increased kidney weight even when related to increased body weight [32]. These animals also develop glomerulosclerosis and kidney failure, in association with glomerular hypertrophy and progressive albuminuria [33]. Transgenic mice overexpressing IGF1 are larger than wild-type mice, have proportionately enlarged kidneys [34], and also show glomerular hyperthrophy, but do not develop glomerulosclerosis [35,36]. These findings indicate that GH excess causes glomerular and podocyte hypertrophy sufficient to induce glomerulosclerosis independently of IGF1.

A study on GH-transgenic IGF1-deficient mice allowed for the demonstration of the dissociated effects of IGF-dependent and independent actions of GH on tubular and glomerular growth in vivo. These mice developed glomerular hypertrophy, hyperplasia, and glomerulosclerosis, similar to GH-transgenic mice with normal IGF1 expression but, in contrast to them, which did not develop proximal tubular cells hyperplasia. These data indicate that IGF1 is not necessary for mediation of the effects of GH-overabundance causing progressive glomerulosclerosis in GH-transgenic mice, but showed that IGF1 is an important mediator of excess GH-induced proximal tubular hyperplasia [37].

Consistent with the role of IGFBPs as inhibitors of IGF action, their generalized overexpression predominantly results in growth retardation. Mice engineered to overexpress IGFBP-1 have prenatal and postnatal growth retardation, disproportionally small brains, splenomegaly, and glucose intolerance. Their kidneys are proportionally small with a decreased nephron number; they also develop glomerulosclerosis without glomerular hypertrophy [38,39,40,41]. Transgenic mice that overexpress IGFBP-2 have only mild growth retardation, with proportionally small kidneys [42]. Mice overexpressing IGFBP-3 have selective organomegaly (spleen, liver, heart) [43], and disproportionally small kidneys [44], whereas those overexpressing a mutant of IGFBP-3 with impaired IGF binding have normal postnatal growth and kidney size [45], suggesting that the effects on the kidney seen in the former are due to inhibition of IGF actions. IGFBP-4 overexpression in various tissues in mice resulted in hypoplasia of the affected tissue, suggesting a common action in different cell types [37]. Interestingly, only few or no phenotypic changes were observed when separately knocking out each specific IGFBP [46,47,48,49,50].

In the 5/6 nephrectomy mouse model of chronic kidney disease, silencing of SOCS2, a negative regulator of GH action, was shown to overcome CKD-related growth retardation without worsening kidney function. This was explained by elevation of inflammatory cytokines in uremic mice and upregulation of SOCS3, another regulator of cytokine signaling, leading to the prevention of renal GHR overstimulation [51].

Additional experiments have shown that most growth-stimulating effects of IGF1 are mediated by its locally produced form, acting in an autocrine or paracrine fashion [14,52]. Inactivation of the liver-specific IGF-1 gene in mice had no effect on somatic growth, demonstrating that local IGF-1 plays an auto/paracrine role in tissues [53].

## 5. GH and IGF1 in Normal Renal Development

The GH/IGF1 system plays a key role in normal kidney development, although it does not impair basic kidney formation mediated by the branching morphogenesis process [54]. During embryogenesis, GHR mRNA was detected in rat kidneys from embryonic day 20 and was mainly expressed in the proximal tubules [55]. In the human fetal kidney, GHR-specific immunostaining was shown as early as 8.5 to 9 weeks and most renal tubular epithelial cells became positive by week 13. The staining was stronger in the outer medulla than in the cortex and remained similar at midgestation and after birth. Weak staining was also found in immature glomeruli in early gestation, but disappeared at later developmental stages, suggesting specific GH involvement in glomerular morphogenesis [56].

IGF1 and -2 are required for normal metanephric development [57]. Studies of IGF1 expression during mouse kidney development revealed IGF1 mRNA expression in all renal cells at embryonic day 15, with a drastic decrease after birth [58].

During early embryogenesis, the IGF1R mRNA is expressed in the rat mesonephros [59] and is detected in all nephron segments through adulthood. In the human kidney, the IGF1R is strongly expressed in glomeruli and the tubular epithelium [60].

IGF-2 plays an important role during embryonic and fetal development, but its function after birth has not been fully elucidated [61]. Transgenic mice overexpressing IGF-2 have disproportionately enlarged kidneys relative to body weight [62].

## 6. GH/IGF1 Effects on Normal Tubular and Glomerular Functions

Normal kidney function includes glomerular filtration and tubular secretion and reabsorption, leading to fluid and electrolyte balance. In addition, kidneys control blood pressure, as well as hormonal synthesis (such as EPO and active Vitamin D).

GH and IGF1 deficient patients have reduced glomerular filtration rate (GFR) and renal perfusion flow (RPF) [63,64]. Hypophysectomy in humans leads to a rapid decrease in GFR [65], and rhGH treatment leads to GFR and RPF improvement in a dose and time-dependent manner [63,64]. In a cohort of GH-deficient children (isolated or multiple pituitary), GFR was in normal physiological levels but lower than in controls and significantly increased after 3 years of rhGH in parallel to kidney and body growth [66]. In contrast, acromegalic patients have increased GFR and RPF [65,67] and albuminuria [68,69,70] compared with healthy subjects.

Evidence on direct actions of IGF1 on glomerular function comes from patients with GH-insensitivity due to GHR mutations, where treatment with rhIGF1 improves GFR [71]. Injection of IGF1 in rodents and humans increases RPF and GFR [72], influencing single-nephron GFR and blood flow by increasing the ultrafiltration coefficient and decreasing efferent arteriolar resistance [73]. This effect depends on the synthesis of endogenous vasodilators including NO and prostaglandins, and can be blocked by inhibition of NO-synthase and COX [74].

Recent studies elucidated the action of IGFs on the glomerular podocyte. IGF-2 action, mediated by the IGF1R, is important for podocyte cell survival and integrity of the glomerular filtrating barrier. Mice with reduced IGF-2 production have abnormal glomeruli, indicating the role of IGF-2 throughout the glomerulus [75].

GH and IGF1 are involved in tubular handling of sodium, water, calcium, and phosphate, and are also known to regulate tubular gluconeogenesis [76]. GH deficiency is associated with reduced sodium and total body water content [77], and rhGH-replacement therapy improves these parameters [78]. Treatment with high rhGH doses may even lead to acute fluid retention [79]. In contrast to that, acromegalic patients show an increase in total body water and sodium and may present with edema. Treatment of GH-producing tumors reverses these changes [80,81].

The direct, IGF-1-independent effect of GH on sodium and fluid retention is controversial: infused recombinant IGF1 did not change body weight and sodium excretion in healthy subjects [18,82], but treatment with rhIGF1 improved hydration status in children with GH insensitivity due to GHR inactivating mutations, indicating that sodium and water retaining properties of GH are at least partly mediated by IGF1 [83].

Liver-specific deletion of the IGF1 gene increased urinary sodium and potassium excretion [84], confirming the role of IGF1 in water and sodium handling. Evidence for both direct GH/IGF1 action on kidney tubule and indirect mechanisms involving the renin-angiotensin-aldosterone system (RAAS) or natriuretic peptides exists. Rapid increase in plasma renin activity and aldosterone level after rhGH administration in healthy men was reported [85], and treatment with angiotensin converting enzyme (ACE)-inhibitor captopril and mineralocorticoid receptor antagonist spironolactone abolished the GH-induced increase in extracellular volume [86]. Decrease of atrial natriuretic peptide concentration after rhGH treatment was also shown [87]. Recent data show evidence for direct action of GH and IGF1 on epithelial sodium channels (controlled by aldosterone) in cortical collecting ducts [88]. Reversal of GH/IGF1 excess in acromegalic patients decreases ENaC activity [89]. In rats with GH-secreting tumors, the direct stimulatory effect of excess GH on ENaC-dependent sodium transport in distal nephron was demonstrated. Enhanced natriuretic response after ENaC blocking by amiloride and enhanced Na/K-ATPase activity selectively in the cortical collecting ducts were demonstrated, providing additional evidence for increased sodium reabsorption in the late distal nephron during a chronic GH excess. Changes in ENaC subunit proteins, known to be associated with increased ENaC activities [90], were shown in these rats and were not accompanied by elevated aldosterone levels [88]. In humans, active acromegaly was also associated with an increased response to amiloride, providing evidence of increased renal ENaC activity in excess of GH/IGF1 [89]. Another possible molecular target of GH/IGF1 in the kidney tubule is the sodium-potassium pump Na/K-ATPase. GH has been shown to enhance the hydrolytic activity of Na/K-ATPase in rat kidney [91].

Being the major hormones mediating somatic growth, GH and IGF1 promote positive calcium and phosphate balance, influencing, for example, 1.25 (OH)_2_ vitamin D synthesis, which is crucial for intestinal calcium absorption. GH stimulation of renal calcitriol synthesis is mediated by IGF1 via induction of 1α-hydroxylase in the proximal tubule [92]. GH-replacement therapy, as well as treatment with rhIGF1, increased serum calcitriol levels in GH-deficient patients [93]. Several studies in GH-deficient adults have shown transient elevation in blood calcium level and urinary calcium excretion during rhGH-treatment [94,95]. In contrast, studies in children showed unchanged or even decreased blood calcium levels during long-term rhGH replacement, probably related to modifications of mineral metabolism and a significant increase in bone density [96].

In adults and children with GHR insensitivity (Laron dwarfism), treatment with rhIGF1 resulted in increases in urinary calcium excretion without changes in serum calcium levels [83,97].

Patients with an excess of GH often have serum calcium concentrations toward the upper-normal range in association with hypercalciuria, which can be consistent with increased calcitriol synthesis [98]. Treatment of healthy subjects, patients with CKD, and GH-deficient patients with rhGH has been shown to increase the circulating levels of sKlotho. Klotho is a co-receptor for the phosphaturic hormone FGF23, which also enhances calcium reabsorption in distal nephron [99,100,101].

Long-term rhGH treatment leads to a persistent increase in plasma phosphate concentrations in GH-deficient children [93] and adults [91,92,102], which is mediated by a direct antiphosphaturic action of IGF1 in the proximal tubule [103]. IGF1 directly increases phosphate reabsorption via increase of Na-Pi2a expression in proximal tubule, which could be completely blocked by an anti-IGF1R antibody [104,105]. Patients with acromegaly may have mild hyperphosphatemia that normalizes after treatment of their GH-secreting tumor [106].

**Figure 1:**
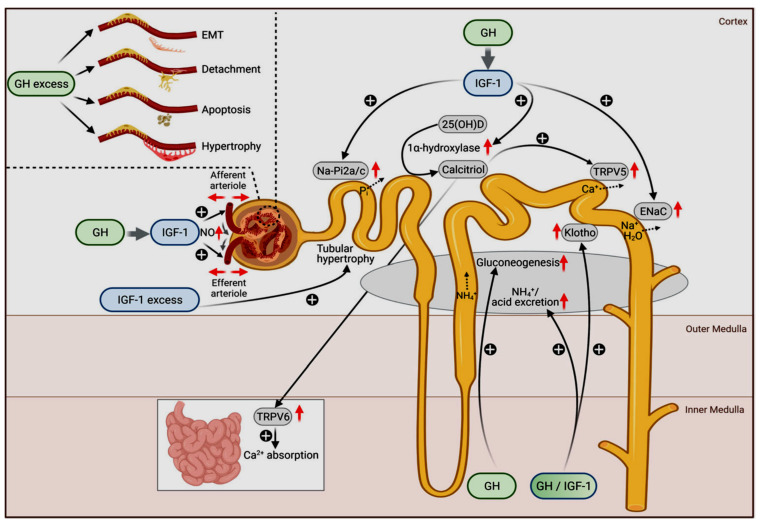
Physiological (main figure) and pathophysiological actions of GH (upper left insert) and IGF-1 on the kidneys. The original figure has been published by Hafner et al. [107] and published here with permission. The figure is licensed under a Creative Commons Attribution 4.0 International License. See link to the Creative Commons license (http://creativecommons.org/licenses/by/4.0/, accessed on 29 November 2021). No changes to the original figure were made.

The physiologic roles of GH and IGF1 in different nephron segments are depicted in Figure 1.

## 7. GH/IGF1 Involvement in Kidney Diseases

### Compensatory Renal Hypertrophy

Following unilateral nephrectomy, the remaining kidney undergoes compensatory growth with an increase in single-nephron GFR and hypertrophy of all nephron segments, especially proximal tubuli. This process is activated by glomerular hemodynamic changes and regulated by positive and negative growth factors [108], including GH and IGF1 in early stages [109,110]. The GH-IGF1 axis is involved in remnant kidney hypertrophy only in early stages, and other mechanisms are involved in the kidney compensatory hypertrophy afterwards [111]. IGF1 was proposed to mediate protein-induced kidney growth. Healthy infants, fed with high-protein formula during the first year of life, showed correlations between IGF1 levels and kidney volume [112].

In adult rats, changes in the pulsatile release of growth hormone (GH), which facilitates compensatory renal growth after unilateral nephrectomy, was observed [113]. Developmental and sex differences in the initial phase of compensatory renal growth following unilateral nephrectomy was shown in animal models. In adult rats, compensatory renal growth was GH-dependent, and GH-independent in immature rats, associated with an increase in local renal IGF-1 and IGF1R mRNA, an effect not seen in adult rats. These age-dependent differences were observed in male rats, but in females compensatory renal growth was associated with increased expression of IGF1 both in juvenile and adult rats, indicating potential gender differences [114].

**Table 1:** Chain of GH-IGF signals: general and kidney phenotypes with loss of function. KO: knockout muse model; NA: not available; m: mouse; h: human.

	KO/Human Mutation General Phenotype	KO/Kidney Phenotype	Ref.
GH	Growth retardation	Disproportionally small kidneys	[17]
GHR/ GHBP	Growth retardation after birth, low IGF1, greater longevity	Disproportionally small kidneys Protection against diabetic nephropathy	[18]
JAK2	Embryonic lethality due to a lack of hematopoiesis	NA	[19]
STAT5	Abnormal postnatal growth, facial dysmorphism, immunodeficiency (h) perinatal death, dwarfism, anemia, immunodeficiency (m)	NA	[20,22]
IGF1	Severe growth retardation, infertility, deficiencies in bone and muscle development, lethal respiratory failure	Proportionally small kidneys, decreased glomerular size and nephron number Liver specific IGF1 KO mice: compensatory remnant kidney hypertrophy after unilateral nephrectomy, no significant change in IGF1R phosphorylation (despite markedly decreased kidney IGF-1 levels)	[23,24,114]
IGF1R	Respiratory failure, low birth weight, developmental abnormalities, perinatal death	NA	[25]
SOCS2	Gigantism, improved somatic growth in CKD model	No glomerulosclerosis development	[47]
IGFBP1	indistinguishable from wild-type, no embryonic lethality	NA	[44]
IGFBP2	minor gender specific changes in bone structure, minor changes in the weights of spleen and liver in adult males	NA	[43,45]
IGFBP3	Normal	NA	[42]
IGFBP4	mild 10%–15% reduction in prenatal growth	NA	[42]
IGFBP5	Normal	NA	[42]
IGFBP6	Normal	NA	[42]

**Table 2:** Effects on general and kidney phenotypes by gain of function in GH-IGF pathway. There are no data about transgenic models for GHR/GHBP, IGF1R, SOCS, and IGFBP5 and -6.

	General Phenotype	Kidney Phenotype	Ref.
GH	Giant phenotype, organomegaly	Kidney hypertrophy, glomerular hyperthrophy, progressive albuminuria, glomerulosclerosis	[27,28,29]
IGF1	Enhanced growth	Proportionately enlarged kidneys, glomerular hyperthrophy, no glomerulosclerosis	[30,31,32]
IGFBP1	Low birth weight, postnatal growth retardation, disproportionally small brain, splenomegaly, hyperglycemia	Small kidneys, decreased nephron number; glomerulosclerosis without glomerular hypertrophy	[34,35,36,37]
IGFBP2	Mild growth retardation, mildly reduced organs weight	NA	[38]
IGFBP3	Increased spleen, liver, heart weight	Disproportionally small kidneys	[38,39,40]
IGFBP4	Different tissues hypoplasia		[37]
IGF2		Disproportionately enlarged kidneys	[58]

GH regulation of angiotensin II receptor 1 (AT1R) expression in the kidney is important for GH-dependent compensatory renal growth in the adult male, but not female, rats. GH suppression abolishes the increase in AT1R expression in remnant kidney in male rats after unilateral nephrectomy [115].

In a knockout mouse model in which the major GH signaling mediator JAK2 was specifically inactivated in the liver, hepatic IGF1 production was demonstrated to be crucial for GH-mediated kidney mass stimulation, suggesting that locally produced renal IGF1 had little or no effect on kidney growth. However, skeletal length was dependent upon or compensated for by locally produced IGF1 [116]. On the other hand, in liver specific IGF1 knockout mice, which showed a major decrease in circulating IGF-1 levels (>90% reduction) and impaired body growth, unilateral nephrectomy induced a significant and proportional increase in renal mass despite markedly decreased kidney IGF-1 levels and no significant change in IGF1R phosphorylation. This suggests that factors other than circulating and locally produced IGF-1 are responsible for compensatory renal enlargement [117].

## 8. Diabetic Nephropathy

Diabetic nephropathy is characterized by glomerular hyperfiltration, glomerular/tubular hypertrophy, thickening of the glomerular basement membrane, and mesangial matrix expansion/proliferation, resulting in increased glomerular permeability, albuminuria, tubulointerstitial fibrosis, and progressive CKD [118]. GH and IGFs play a significant role in the early development of diabetic renal disease [119]. Increased GH secretion with decreased expression of GHR in the liver and decreased serum IGF1, consistent with the GH resistance, was shown in diabetic mice as well as in patients with uncontrolled diabetes mellitus [120,121]. As previously mentioned, excessive levels of GH induce glomerular hypertrophy and glomerulosclerosis [32,33].

In diabetic rats, GH treatment exacerbated the course of diabetic renal disease [122]. In contrast, GH-deficient rats are relatively protected from diabetic related renal hypertrophy [123]. GH antagonist administration to non-obese diabetic mice inhibited early diabetic glomerular hyperfiltration, hypertrophy, and albuminuria [124]. Treatment with GH antagonist in T1DM patients resulted in significant reduction in kidney volume and hyperfiltration [125]. The effects of somatostatin analogs on nephropathy in type 1 diabetes are comparable to the effect of angiotensin-converting enzyme inhibitor treatment [126].

Reduced circulating IGF1 was reported in T1DM patients, while renal IGF1 concentration was increased in an animal diabetic model, suggesting increased local synthesis [127]. Increased IGF1R and IGFBP in renal tissue was found in the early course of diabetic nephropathy in experimental models as well [128,129].

The early increase in IGF1 leads to the rise in GFR by reducing renal arteriolar resistance and increases the glomerular ultrafiltration coefficient, as previously mentioned [72].

GHR and IGF1R are highly abundant in glomerular cells, including podocytes and mesangial cells. Podocyte hypertrophy, apoptosis, dedifferentiation due to epithelial-to- mesenchymal transition, and detachment from the glomerular basement membrane were shown to be early events in the development of diabetic nephropathy in humans and various animal models of diabetic nephropathy [130]. GH increases levels of reactive oxygen species and induces actin cytoskeleton reorganization in podocytes, causing abnormal functioning of the slit diaphragm, increased permeability of the filtration barrier, and albuminuria [131]. Mesangial cells isolated from experimental models of diabetic nephropathy exhibit altered IGF1 synthesis, IGF1 pathway activation, and higher IGF1R expression and activation compared with controls [132]. Hyperglycemia reduces IGFBP-2 expression in mesangial cells, exacerbating IGF1 effects on mesangial cells, and increases the expression of IGFBP-3, which mediates mesangial cell apoptosis [133,134].

Several mechanisms have been proposed to explain the role of GH and IGF1 in diabetic nephropathy. GH stimulates the expression of transforming growth factor-beta-induced protein (TGFBIp) in cultured podocytes, increasing podocyte migration and permeability of podocyte layer to albumin [135]. TGFBIp was found to be upregulated in renal tissue from patients with diabetic nephropathy, suggesting GH induction of TGFBIp, which may contribute to podocyte depletion in diabetes mellitus.

Using cultured immortalized podocytes and mouse models, it was demonstrated that GH excess activates Notch1 signaling in podocytes, resulting in podocyte loss. GH-induced glomerular fibrosis, glomerular basement membrane thickening, and albuminuria in vivo were prevented by pharmacological inhibition of Notch1 [136]. Upregulated Notch signaling was also noted in kidney biopsies from patients with diabetic nephropathy.

IGF1 effects in the kidney are modulated by nitric oxide, and nitric oxide synthase inhibition reduced renal hypertrophy and hyperfiltration in STZ-induced diabetes mellitus rats [137]. Prevention of advanced glycation end products accumulation in the STZ-induced model of diabetes has been reported to inhibit overexpression of IGF1, IGFBP-1, and IGFBP-4 mRNAs, suggesting the role of glycation end products in activation of IGF1 renal expression in diabetes mellitus [138]. In the diabetic rat model, insulin inhibits IGF1’s action on glomeruli by upregulation of the STAT5/SOCS2 pathway. STAT proteins activate gene transcription of IGF1, and SOCS proteins inhibit it. Thus, downregulation of this pathway in mesangial cells in insulin deficiency leads to increased actions of IGF1 on matrix production, glomerular enlargement, and progression of diabetic nephropathy [139].

## 9. Chronic Kidney Disease (CKD)

CKD is defined as permanent kidney damage, structural or functional, with or without a decrease in glomerular filtration rate (GFR). CKD is divided into five stages according to the decrease in estimated GFR. The nature of CKD is progressive in most patients, in association with many complications. CKD progression is related to many signaling pathways, mostly the renin-angiotensin-aldosterone system. CKD and its complications in children are associated with alterations in the GH/IGF1 axis, including growth retardation. GH levels in CKD are slightly elevated due to its impaired renal clearance, prolonged half-life, and the state of GH resistance. Renal GH resistance results from reduced GH receptor numbers in target tissues, post-receptor defects in GH signaling, and reduced levels of free IGF1 [140].

Reduced expression of the GH receptor has been shown in the epiphyseal growth plate of uremic rats [141]. GHBP is reduced in CKD proportionally to the degree of renal dysfunction [142]. Impaired postreceptor GH-activation of the JAK2 signaling pathway and downstream phosphorylation of STAT proteins and overexpression of SOCS, an inhibitor of JAK2/STAT5 signal transduction and GHR, also results in CKD-associated GH resistance [143]. Elevated expressions of SOCS proteins mRNA in skeletal muscle, liver, and epiphyseal growth plate have been found in uremic rats [144,145,146]. Impaired equilibrium between GHR-JAK2-STAT signaling and SOCS expression has also been described in chronic inflammation [140]. Proinflammatory cytokine IL-6, its signaling protein STAT3, and its gene product SOCS-3 were found to be significantly increased in uremia. SOCS-3 is a potent negative feedback inhibitor of GH signaling and may contribute to the GH-resistant state in CKD [147].

There is also evidence for IGF1 insensitivity in CKD [148]. Serum total IGF1 concentration is normal in patients with CKD, but reduced bioavailability is related to inhibitory effects of IGFBPs, which levels are increased in CKD. Both decreased renal clearance and increased hepatic production contribute to accumulation of IGFBPs in uremia [149]. In addition, impaired cellular IGF signaling was demonstrated in experimental uremia [150]. GH treatment in CKD increases serum IGF1 levels and alters the balance of IGFBPs, resulting in a marked increase in IGF1 bioactivity [151].

CKD complications also contribute to GH/IGF1 axis alteration. Metabolic acidosis inhibits pituitary GH secretion and down-regulates hepatic IGF1 and GH receptor mRNA expression [152]. Long-term steroid therapy affects pulsatile GH secretion and inhibits hepatic production of IGF1 [153].

As discussed previously, GH increases GFR and RPF, and leads to glomerulosclerosis in transgenic mice overexpressing GH, raising concern about adverse effects of rhGH treatment on CKD progression. Indeed, in subtotally nephrectomized rats, treatment with high doses of rhGH resulted in a high glomerular sclerosing index, but low rhGH-dose did not result in significant changes in GFR or glomerular sclerosis index compared to controls [154]. Other animal studies did not show significant changes in GFR and RPF after treatment with bovine GH and rhIGF1 in 5/6 nephrectomized rats. In contrast, healthy rats showed an increase in GFR and RPF after treatment with bovine GH and rhIGF1 [155]. Clinical studies of rhGH therapy for short stature in CKD do not support preclinical findings on kidney function deterioration obtained in animals. Treatment with pharmacologic doses of rhGH did not increase GFR in patients with CKD 3–4 [156,157]. Recent experimental studies suggest that the safety of prolonged rhGH-treatment in CKD may be explained by a GH-resistant state in CKD [51].

## 10. Summary

The GH/IGF-1 axis plays a significant role in kidney growth, physiology, and pathophysiology. Its main actions become apparent in pathophysiologic situations of GH excess or deficiency, and the pathophysiological pathways behind these clinical observations were clarified with the development of transgenic and knockout animal models. GH and IGF-1 are involved in regulation of glomerular hemodynamics and tubular handling of water, sodium, calcium, and phosphorus. They participate in pathophysiology of diabetic nephropathy, as well as in compensatory kidney hypertrophy after unilateral nephrectomy. Alterations in GH/IGF-1 system are involved in pathogenesis of growth retardation in children with CKD. Studies on GH-IGF1 axis in CKD and diabetic nephropathy showed that systemic levels of GH and IGF1 do not always reflect their local levels and actions in the kidney. Treatment with rhGH for short stature in CKD was shown to be safe regarding the lack of impairment in renal function deterioration, which can probably be explained by a significant kidney GH resistance state in CKD.

## Data Availability

Not applicable.
